# Gender difference in the relationship between serum uric acid reduction and improvement in body fat distribution after laparoscopic sleeve gastrectomy in Chinese obese patients: a 6-month follow-up

**DOI:** 10.1186/s12944-018-0934-y

**Published:** 2018-12-20

**Authors:** Xuane Zhang, Cuiling Zhu, Jingyang Gao, Fangyun Mei, Jiajing Yin, Le Bu, Xiaoyun Cheng, Chunjun Sheng, Shen Qu

**Affiliations:** 1 Department of Endocrinology and Metabolism, Shanghai Tenth People’s Hospital, Tongji University School of Medicine, No.301 Middle Yanchang Road, Shanghai, 200072 China; 2 National Metabolic Management Center (Shanghai 10th People’s Hospital), Shanghai, 200072 China; 3 Department of Endocrinology and Metabolism, YangPu Hospital, Tongji University School of Medicine, Shanghai, 200090 China

**Keywords:** Serum uric acid, Body fat distribution, Gender difference, Obesity,Laparoscopic sleeve gastrectomy

## Abstract

**Background:**

Hyperuricemia is related to obesity and fat accumulation. This study aimed to observe the effects of laparoscopic sleeve gastrectomy (LSG) on serum uric acid (sUA) level and body fat distribution in obese patients. The relationships between post-LSG improvement in sUA levels and body fat distribution changes, as well as their sex-related differences, were also explored.

**Methods:**

In total, 128 obese patients (48 men; 80 women) who underwent LSG were enrolled. Anthropometric indicators, glucose and lipid metabolic indicators, and sUA levels were measured pre-LSG and 6 months post-LSG. The body compositions were measured via dual-energy X-ray absorptiometry. The patients were divided into normal-sUA (NUA) and high-sUA (HUA) groups based on preoperative sUA levels.

**Results:**

Compared with the NUA group, the reduction of sUA levels 6 months post-LSG was more significant in the HUA group. In addition, sUA reduction in the female HUA group was more significant than that of the male HUA group (*P <* 0.01). Changes in serum uric acid levels (ΔsUA) in the male HUA group was positively correlated with changes in body weight, body mass index, neck circumference, and hip circumference (*r* = 0.618, 0.653, 0.716, and 0.501, respectively; *P <* 0.05 in all cases). It was also positively correlated with changes in fat mass in the gynoid region, android region, and legs, (*r* = 0.675, 0.551, and 0.712, respectively; *P <* 0.05 in all cases), and negatively correlated with changes in total testosterone (ΔTT) (*r* = − 0.517; *P =* 0.040). Furthermore, ΔTT in this group was closely associated with the improved sex-related fat distribution. The ΔsUA in the female HUA group was positively correlated with changes in fasting serum C peptide and ΔLNIR (*r* = 0.449 and 0.449, respectively; *P <* 0.05 in both cases). In addition, it was also positively correlated with changes in visceral adipose tissue (VAT) fat mass, VAT fat volume, and VAT fat area (*r* = 0.749, 0.749, and 0.747, respectively; *P <* 0.01 in all cases).

**Conclusions:**

sUA levels of obese patients with hyperuricemia improved 6 months after LSG. Reduction of sUA after LSG was correlated with improved body fat distribution, and the relationships also displayed sex-based differences. Uric acid might be an important metabolic regulator associated with fat distribution and sex hormones.

## Background

The prevalence of obesity has been increasing every year due to changes in lifestyle and dietary patterns in the population. Elevated serum uric acid (sUA) levels are a common comorbidity of obesity [1]. Hyperuricemia is a disease characterized by an abnormal increase in sUA level in the human body due to aberrant purine metabolism. Recent studies have shown that uric acid is not only the product of purine metabolism but may also have a role similar to cytokines in that it promotes inflammation and participates in the development of obesity. Hyperuricemia may be improved by controlling body weight. Bariatric surgery is currently the only effective option to achieve long-term stable weight loss in obese patients [2]. Laparoscopic sleeve gastrectomy (LSG) is an important bariatric surgery used in the treatment of patients with morbid obesity [3]. Clinical studies have shown that in addition to effectively reducing body weight in obese patients, LSG can also improve body fat distribution and relieve hyperuricemia [4]. However, the exact mechanisms of these effects remain poorly understood. The purpose of this study was to observe the effects of LSG on sUA levels and body fat distribution in obese patients through follow-ups. The correlation between sUA levels and body fat distribution was also investigated. This study further explored LSG’s effects on sUA levels and fat distribution improvement in male and female populations to provide insight on the mechanisms of bariatric surgery in ameliorating hyperuricemia.

## Methods

### Patients

Obese patients admitted to the Tenth People’s Hospital of Tongji University between August 2012 and July 2017 who underwent LSG were selected. We included patients with a body mass index (BMI) ≥ 30 kg/m^2^ and who successfully underwent LSG with regular follow-ups for 6 months. Patients with the following characteristics were excluded from the study: secondary obesity due to endocrine disorders, history of malignant tumors, severe hepatic and renal dysfunction, presence of cardiocerebral vascular disease, previous use of glucocorticoids, niacin, or uric acid-lowering drugs, concurrent participation in other clinical trials, severe endocrine and hereditary diseases, and mental illnesses that rendered them unable to provide informed consent. This study was approved by the Ethical Committee of the Shanghai Tenth People’s Hospital. All clinical data and physical examination data were collected with the consent of patients and their families (registration number: ChiCTR-OCS-12002381). Based on their sUA levels, the patients were divided into normal sUA (NUA) and high sUA (HUA). The NUA group included men with sUA < 420 μmol/L and women with sUA < 360 μmol/L. The HUA group included men with sUA ≥ 420umol/L and women with sUA ≥ 360 μmol/L.

### Anthropometric assessment and laboratory analysis

The height (H), body weight (BW), neck circumference (NC), waist circumference (WC), and hip circumference (HC) were measured by trained specialists pre-LSG and 6 months post-LSG. We calculated body mass index (BMI) and waist hip ratio (WHR) as follows: BMI=BW/H*H(kg/m^2^) and WHR = WC/HC.

Fasting venous blood samples were taken for determining the level of fasting plasma glucose (FPG), fasting serum insulin (FINS), and fasting serum C peptide (FCP). Hemoglobin Alc (HbAlc) was detected by high performance liquid chromatography. Triglyceride (TG), total cholesterol (TC), high-density lipoprotein cholesterol (HDL), low-density lipoprotein cholesterol (LDL), and sUA levels were determined using enzymatic assays. Levels of sex hormones such as estradiol (E2), total testosterone (TT), follicle-stimulating hormone (FSH), and luteinizing hormone (LH) were measured using radioimmunoassay. The homeostasis model assessment insulin resistance index (HOMA-IR) was then calculated using the following formula: FPG(mmol/L)*FINS (mU/L)/22.5. The ratio of fasting plasma glucose and fasting serum insulin (FGIR) was calculated as FPG(mg/dl)/FINS(mU/L), and the ratio of postoperative uric acid reduction was calculated as (sUA_preoperative_ - sUA_postoperative_)/sUA_preoperative_.

### Measurement of body composition

Dual-energy X-ray absorptiometry (DEXA, APEX4.5.0.2, HOLOGIC, USA) was used to measure the compositions of various body parts. The fat mass, fat amount, and lean mass were measured in the whole body and in six different regions including arms, legs, trunk, head, android and gynoid regions. Android and gynoid were used to represent two main types of fat distribution. Android mainly referred to body fat around the abdomen. Gynoid referred to body fat around the buttocks and thighs. The android/gynoid fat ratio, trunk/legs fat ratio, and trunk/limbs fat ratio were calculated. The visceral adipose tissue (VAT) fat mass, VAT fat volume, and VAT fat area were calculated using the DEXA software.

### Statistical analysis

Statistical analyses were performed using SPSS 20.0 software (Chicago, IL, USA). Continuous variables with a normal distribution are expressed as means ± SDs and categorical variables are presented as percentages. Non-normally distributed data were logarithmically transformed to normality (HOMA-IR) when needed. The Student’s t-test was used, as appropriate, to determine differences in continuous variables. Paired sample t-tests were used to compare pre- and post-operative levels of relevant indicators. Pearson’s correlation coefficient analysis was used to analyze the correlations between changes in pre- and postoperative sUA levels and body fat changes, as well as related metabolic indicator changes (differences in values were represented by △). Two-sided *p*-value of *<* 0.05 was considered statistically significant in all tests.

## Results

### Baseline characteristics and comparison of anthropometric and biochemical indicators pre- and post-LSG

Among the 128 obese patients who underwent LSG surgery, 40 (13 men and 27 women) were assigned to the NUA group and 88 (35 men and 53 women) were assigned to the HUA group. Among the 128 obese patients, the mean age was 32.23 ± 10.52 years, preoperative weight was 112.92 ± 22.92 kg, and BMI was 39.66 ± 6.23 kg/m^2^. Baseline characteristics and biochemical indicators are shown in Table 1.

**Table 1:** Anthropometric and metabolic characteristics of the patients in normal UA group, high UA group and total patients at baseline and at 6 months after LSG

Characteristic	NUA (*n* = 40)		HUA (*n* = 88)		Total patients (*n* = 128)	
	0 month	6 months	0 month	6 months	0 month	6 months
Gender (Make/Female)	13/27	/	35/53	/	48/80	/
Age (years)	35.10 ± 12.77	/	30.93 ± 9.11	/	32.23 ± 10.52	/
BW (kg)	111.63 ± 21.41	81.37 ± 16.24**	113.51 ± 23.68	86.37 ± 19.12**	112.92 ± 22.92	84.73 ± 18.24**
BMI (kg/m^2^)	39.75 ± 6.32	28.63 ± 4.23**	39.62 ± 6.22	29.53 ± 4.44**	39.66 ± 6.23	29.24 ± 4.35**
NC (cm)	42.42 ± 4.27	37.44 ± 3.73**	43.38 ± 4.80	38.61 ± 4.26**	43.07 ± 4.64	38.23 ± 4.10**
WC (cm)	122.06 ± 15.19	94.97 ± 12.34**	119.93 ± 14.55	97.18 ± 11.98**	120.59 ± 14.73	96.47 ± 12.03**
HC (cm)	122.43 ± 11.41	101.55 ± 9.51**	121.61 ± 11.84	105.21 ± 9.41**	121.87 ± 11.66	104.03 ± 9.51**
WHR	1.00 ± 0.06	0.93 ± 0.05**	0.98 ± 0.08	0.92 ± 0.06**	0.99 ± 0.07	0.92 ± 0.06**
SBP (mmHg)	132.65 ± 14.44	112.23 ± 11.78**	135.02 ± 15.93	123.67 ± 12.23**	134.27 ± 15.45	120.07 ± 13.12**
DBP (mmHg)	84.02 ± 9.90	68.00 ± 12.20**	84.63 ± 10.83	78.81 ± 8.88**	84.44 ± 10.51	75.40 ± 11.14**
sUA (umol/L)	324.63 ± 44.09	317.77 ± 65.24	467.26 ± 82.02b	410.48 ± 93.32b**	422.69 ± 98.03	381.21 ± 95.35**
Glucose metabolism						
FPG(mmol/L)	5.95 ± 1.33	4.65 ± 0.86**	5.98 ± 1.80	4.77 ± 0.73**	5.97 ± 1.67	4.73 ± 0.77**
FINS (mIU/L)	32.08 ± 22.17	8.33 ± 3.62**	34.39 ± 22.21	11.88 ± 5.93b**	33.67 ± 22.13	10.71 ± 5.52**
FCP (mmol/L)	4.22 ± 1.97	2.27 ± 0.62**	4.71 ± 1.60	2.56 ± 0.80**	4.55 ± 1.73	2.46 ± 0.75**
HOMA-IR	9.25 ± 6.78	1.68 ± 0.69**	9.13 ± 6.50	2.61 ± 1.62b**	9.17 ± 6.56	2.30 ± 1.44**
LNIR	0.86 ± 0.30	0.18 ± 0.19**	0.87 ± 0.26	0.33 ± 0.28a**	0.87 ± 0.27	0.28 ± 0.26**
FGIR	4.75 ± 2.84	12.54 ± 7.76**	4.44 ± 4.48	9.76 ± 7.26**	4.52 ± 4.07	10.67 ± 7.48**
HBA1c(%)	6.33 ± 1.29	5.35 ± 0.62**	6.23 ± 1.20	5.26 ± 0.52**	6.26 ± 1.22	5.29 ± 0.55**
Lipid metabolism						
TG (mmol/L)	1.75 ± 1.54	0.81 ± 0.31	2.14 ± 2.36	0.99 ± 0.49**	2.02 ± 2.14	0.93 ± 0.44**
TC (mmol/L)	4.34 ± 1.01	4.24 ± 0.99	4.68 ± 0.99	4.52 ± 0.91	4.57 ± 1.01	4.43 ± 0.94
LDL (mmol/L)	2.61 ± 0.77	2.61 ± 0.98	2.93 ± 0.87	2.87 ± 0.75	2.83 ± 0.85	2.79 ± 0.83
HDL (mmol/L)	1.03 ± 0.28	1.17 ± 0.18**	1.00 ± 0.19	1.20 ± 0.24b**	1.01 ± 0.22	1.19 ± 0.22**

Data presented as mean ± SD; Compare HUA group to NUA group at baseline, ^a^ *P* < 0.05, ^b^ *P* < 0.01; Compare group after 6 months to baseline *P < 0.05, **P < 0.01

At baseline, BW, BMI, NC, WC, HC, WHR, systolic blood pressure (SBP), diastolic blood pressure (DBP), FPG, FINS, FCP, HBA1c, HOMA-IR, FGIR, TG, TC, LDL, and HDL levels were not statistically different between the NUA and HUA groups. At 6 months after surgery, the levels of BW, BMI, NC, WC, HC, WHR, SBP, DBP, FPG, FINS, FCP, HBA1c, HOMA-IR, and HDL in the 2 groups were significantly lower when compared with preoperative levels (*P <* 0.01 in all cases). FGIR was significantly increased after surgery (*P <* 0.01, in all groups). Postoperative TG levels were significantly decreased in the HUA group, whereas no statistical differences between pre- and postoperative TG levels were observed in the NUA group (*P >* 0.05). There was no statistical difference in changes between pre- and postoperative TC and LDL levels, respectively.

**Fig. 1:**
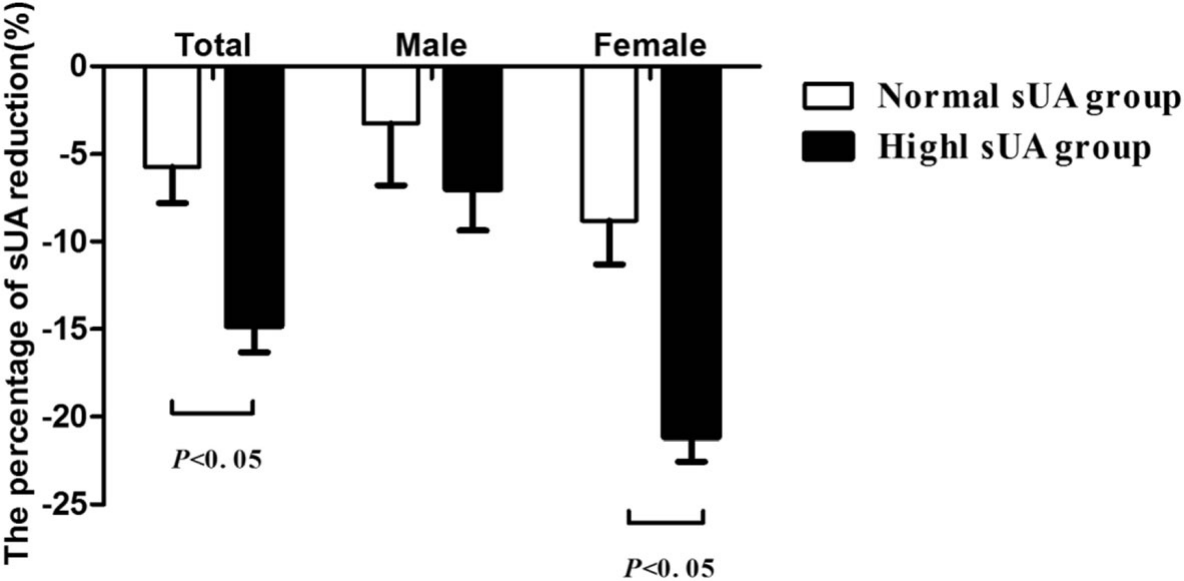
Comparing sUA reduction in HUA and NUA groups after LSG. Data are presented as mean. Error bars are SEM

The baseline sUA level was significantly higher in the HUA group than in the NUA group (*P <* 0.01). The sUA levels decreased in both groups 6 months after surgery. The percentage of sUA reduction was more pronounced in the HUA group than in the NUA group, with a significant difference in the reduction of postoperative sUA between the 2 groups (*P <* 0.05) as shown in Fig. 1.

### Effects of LSG on body fat distribution in obese patients

There were no significant differences in preoperative body fat mass and lean mass distribution between the obese patients in the NUA and HUA groups (*P >* 0.05). Following LSG, the amount of fat mass and lean mass was significantly decreased in various body parts, and this difference was statistically significant (*P <* 0.05, Table 2). The amount of minerals in each body part did not change significantly before and after surgery (*P >* 0.05, data not listed in the table).

**Table 2:** Body composition and derived indexes in HUA, NUA and total patients at baseline and at 6 months after LSG

Variable		NUA group (*n* = 40)		HUA group(*n* = 88)		Total patients (*n* = 128)	
		0 month	6 months	0 month	6 months	0 month	6 months
Total	Fat mass (kg)	49.36 ± 11.90	31.37 ± 7.16**	48.80 ± 10.30	32.68 ± 8.29**	48.90 ± 10.80	32.24 ± 7.88**
	Lean mass (kg)	55.00 ± 10.94	45.13 ± 10.85**	56.79 ± 12.23	50.12 ± 10.75**	56.20 ± 11.81	48.46 ± 10.92**
	Fat% (%)	46.07 ± 6.27	40.00 ± 6.11**	45.12 ± 4.91	38.18 ± 4.88**	45.43 ± 5.38	38.78 ± 5.33**
Head	Fat mass (kg)	1.72 ± 0.47	1.37 ± 0.24**	1.72 ± 0.35	1.82 ± 2.19**	1.72 ± 0.40	1.41 ± ±0.27**
	Lean mass (kg)	4.38 ± 0.72	3.89 ± 0.62**	4.48 ± 0.74	4.02 ± 0.63**	4.45 ± 0.73	3.98 ± 0.62**
	Fat% (%)	25.37 ± 2.54	23.30 ± 0.91**	25.29 ± 1.59	25.14 ± 9.28**	25.31 ± 1.94	23.44 ± 1.31**
Arms	Fat mass (kg)	6.61 ± 1.80	4.41 ± 1.24**	6.93 ± 1.98	4.38 ± 1.10**	6.83 ± 1.92	4.39 ± 1.13**
	Lean mass (kg)	5.08 ± 1.61	4.45 ± 1.65	5.43 ± 1.64	5.03 ± 1.47**	5.31 ± 1.63	4.83 ± 1.54**
	%FM (%)	54.93 ± 9.61	48.79 ± 9.03**	54.67 ± 8.31	45.32 ± 7.67**	54.75 ± 8.71	46.48 ± 8.22**
Legs	Fat mass (kg)	13.75 ± 4.17	9.01 ± 2.42**	14.04 ± 4.28	9.49 ± 2.88**	13.94 ± 4.22	9.33 ± 2.72**
	Lean mass (kg)	17.66 ± 4.13	14.32 ± 3.67**	19.85 ± 10.80	16.36 ± 3.93**	19.14 ± 9.21	15.68 ± 3.93**
	%FM (%)	42.31 ± 7.26	37.46 ± 7.04**	41.00 ± 7.51	35.31 ± 6.78**	41.42 ± 7.42	36.02 ± 6.87**
Trunk	Fat mass (kg)	27.27 ± 7.09	16.57 ± 4.11**	25.98 ± 5.64	17.39 ± 4.85**	26.40 ± 6.15	17.11 ± 4.58**
	Lean mass (kg)	27.74 ± 5.59	22.45 ± 5.32**	28.07 ± 6.01	24.70 ± 5.16**	27.96 ± 5.85	23.95 ± 5.27**
	%FM (%)	48.67 ± 6.19	41.89 ± 6.32**	47.48 ± 4.64	40.33 ± 4.82**	47.87 ± 5.20	40.85 ± 5.35**
Android	Fat mass (kg)	5.11 ± 1.31	2.76 ± 0.91**	4.78 ± 1.21	2.85 ± 0.91**	4.89 ± 1.25	2.82 ± 0.90**
	Lean mass (kg)	4.51 ± 1.06	3.48 ± 1.01**	4.51 ± 1.03	3.66 ± 0.91**	4.51 ± 1.04	3.60 ± 0.93**
	%FM (%)	52.93 ± 5.46	44.06 ± 6.72**	51.44 ± 3.93	43.46 ± 4.91**	51.93 ± 4.52	43.65 ± 5.50**
Gynoid	Fat mass (kg)	6.76 ± 1.66	4.23 ± 1.07**	6.69 ± 1.84	4.51 ± 1.25**	6.71 ± 1.77	4.42 ± 1.19**
	Lean mass (kg)	8.92 ± 1.69	7.08 ± 1.76**	9.07 ± 2.14	7.88 ± 1.80**	9.02 ± 2.00	7.62 ± 1.81**
	%FM (%)	43.01 ± 7.26	37.80 ± 7.71**	42.34 ± 6.35	36.40 ± 6.27**	42.55 ± 6.64	36.85 ± 6.72**
VAT	Vat mass (kg)	1.31 ± 0.34	0.66 ± 0.17**	1.20 ± 0.30	0.71 ± 0.21**	1.23 ± 0.31	0.70 ± 0.19**
	Vat volume (cm3)	1432.11 ± 361.52	713.53 ± 184.42**	1299.59 ± 320.30	776.80 ± 227.31**	1340 ± 337.68	756.17 ± 214.31**
	Vat area (cm2)	271.54 ± 69.89	137.102 ± 35.38**	249.30 ± 61.43	148.83 ± 43.52**	256.57 ± 64.84	145.01 ± 41.02**
Indexes	A/G	1.22 ± 0.13	1.18 ± 0.14*	1.21 ± 0.15	1.21 ± 0.19*	1.23 ± 0.14	1.20 ± 0.17**
	Trunk/legs	1.15 ± 0.11	1.12 ± 0.10*	1.17 ± 0.15	1.16 ± 0.18*	1.16 ± 0.14	1.15 ± 0.15**
	Trunk/limbs	1.36 ± 0.27	1.22 ± 0.19*	1.30 ± 0.29	1.26 ± 0.25*	1.32 ± 0.29	1.25 ± 0.23**

Data presented as means ± SD; Compare group after 6 months to baseline *P < 0.05, **P < 0.01

**Fig. 2:**
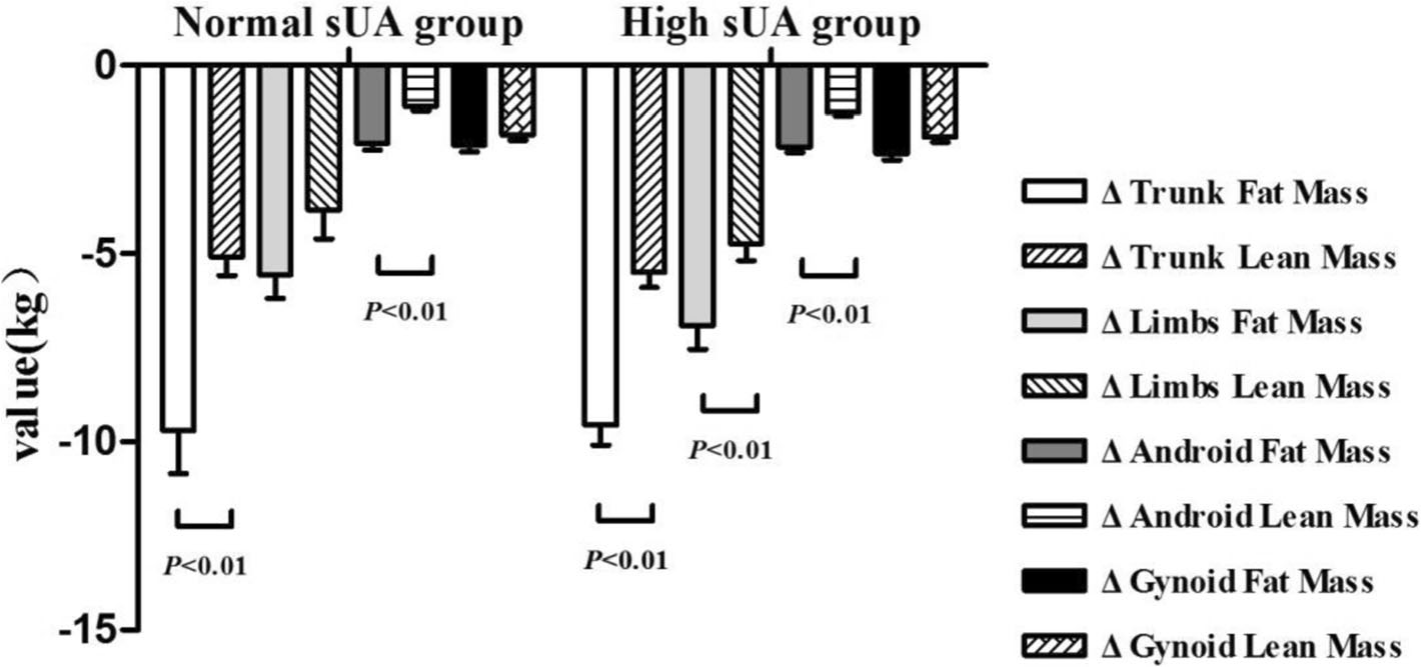
Comparing changes in fat mass and lean mass in HUA and NUA groups after LSG. Data are presented as mean. Error bars are SEM

Additional analysis revealed that reduction in fat mass in the trunk, limbs, and the android region after LSG was more significant compared to reduction in lean mass in the same areas. This observation was particularly evident in the HUA group (*P <* 0.05 in all cases). The reduction in body fat after surgery was mainly due to reduced body fat mass in the trunk (Fig. 2).

**Fig. 3:**
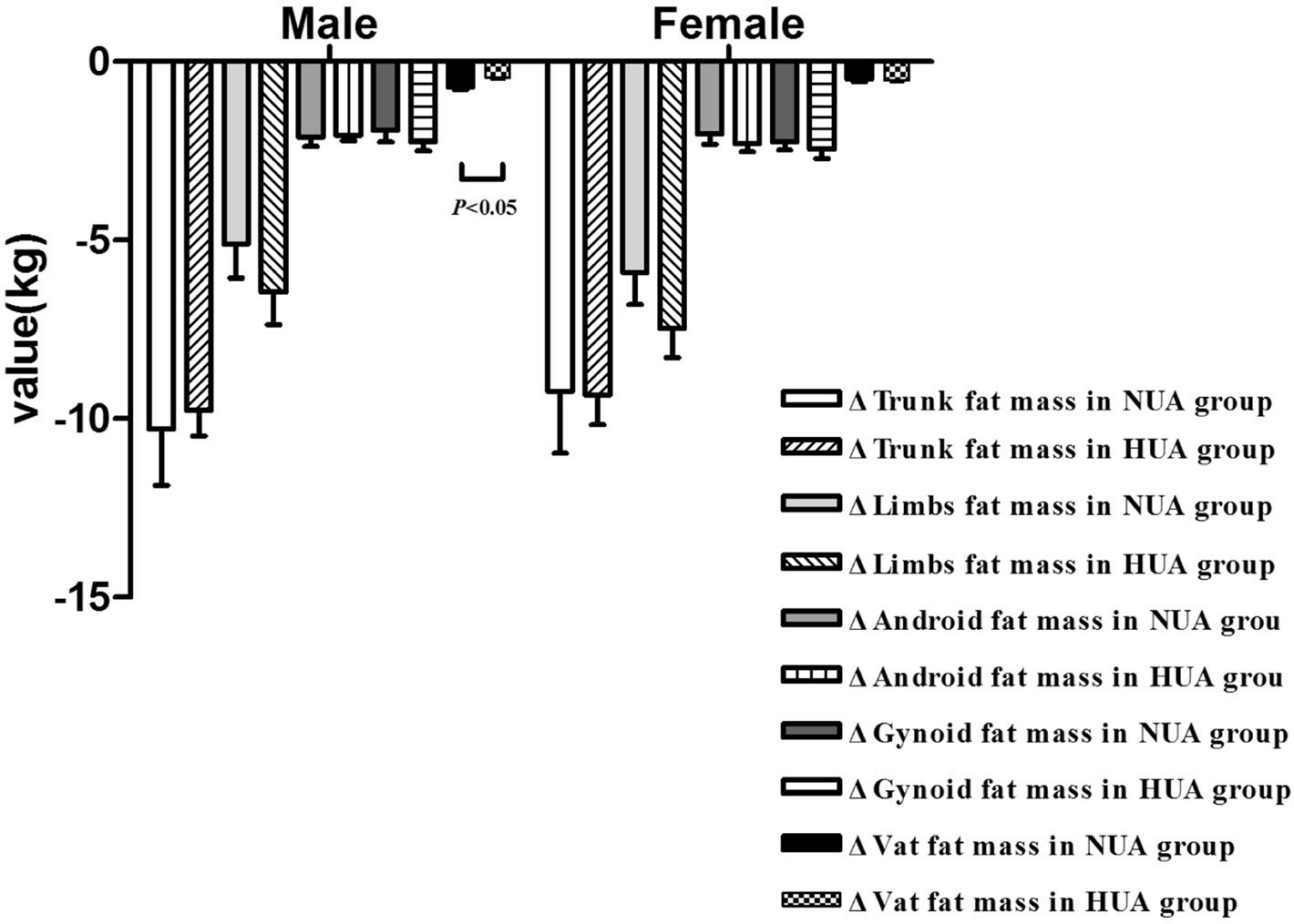
A Comparison of different part in fat mass after LSG in men and women. Data are presented as mean. Error bars are SEM

Furthermore, data grouped based on gender (Fig. 3) showed that the reduction in ΔVAT fat mass in the male NUA group was significantly more pronounced than the reduction in ΔVAT fat mass in the male HUA group (*P <* 0.05), whereas the reduction in fat mass in various body parts was not significantly different between the female NUA and HUA groups (*P >* 0.05).

### Correlation between changes of serum uric acid level (ΔsUA) and anthropometric indicators, metabolic indicators, insulin resistance, and body fat distribution

At baseline, the sUA levels in the HUA group were positively correlated with BW, BMI, NC, WC, and HC (*r* = 0.482, 0.367, 0.486, 0.370, and 0.313, respectively; *P <* 0.001 in all cases) and positively correlated with FCP (*r* = 0.248; *P =* 0.021). At 6 months after LSG, the ΔsUA in the HUA group was positively correlated with ΔFBG (*r* = 0.354; *P =* 0.027) and ΔLNIR (*r* = 0.436; *P =* 0.006). There was also a positive correlation between ΔsUA and ΔBW, ΔBMI, ΔNC, ΔWC, and ΔHC (*r* = 0.347, 0.477, 0.449, 0.373, and 0.466, respectively; *P <* 0.05 in all cases) in the HUA group. Additionally, analysis of the correlation between ΔsUA and body fat distribution revealed that ΔsUA in the HUA group was positively correlated with ΔTotal fat mass, ΔGynoid fat mass, ΔAndroid fat mass, ΔArms fat mass, ΔVAT fat mass, ΔVAT fat volume, and ΔVAT fat area (*r* = 0.410, 0.449, 0.484, 0.396, 0.637, 0.637, and 0.638, respectively; *P <* 0.05 in all cases).

**Fig. 4:**
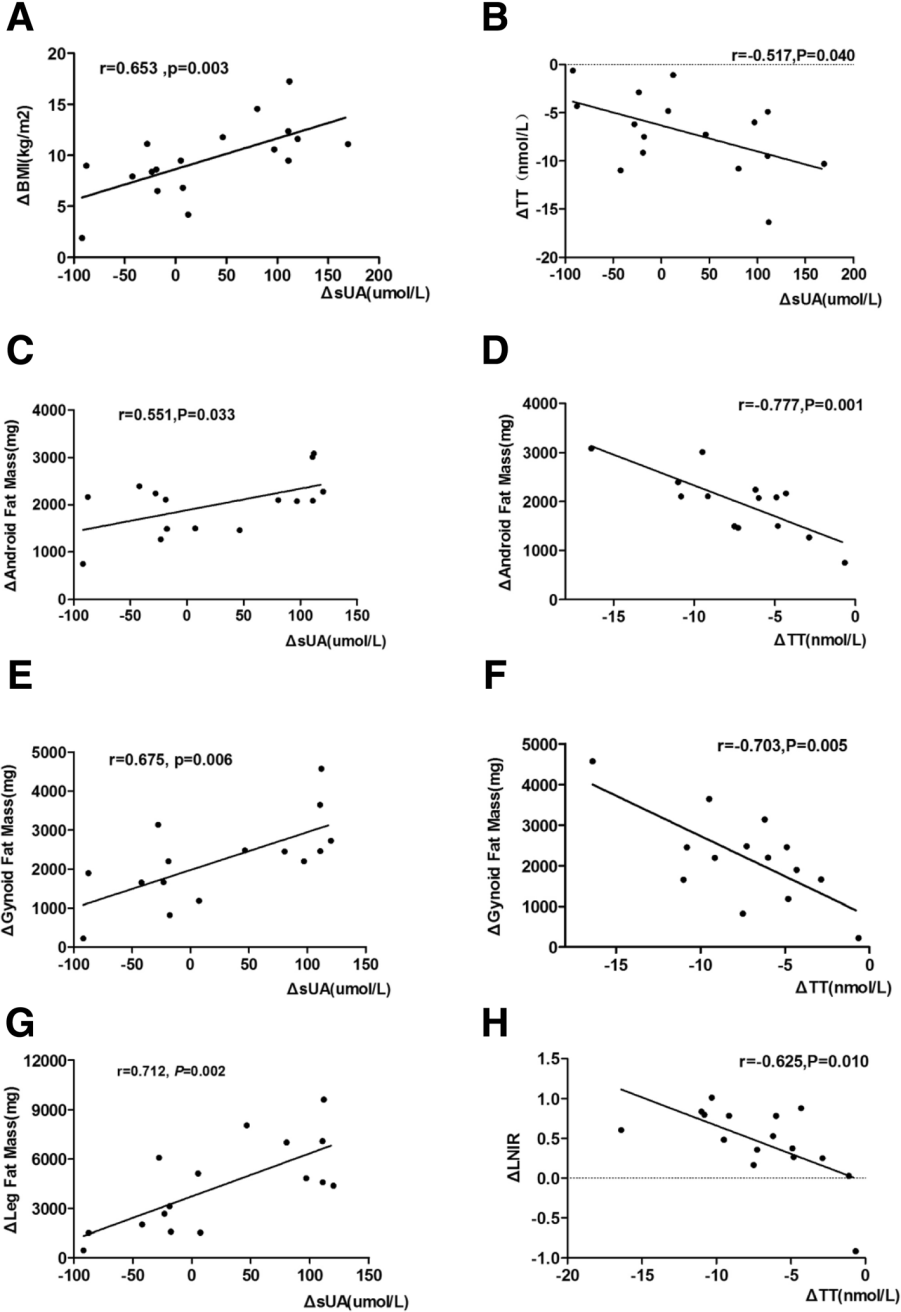
Correlations between ΔsUA and BMI, body fat mass, and TT changes in male HUA group. Changes in sUA were positively associated with changes in BMI (**a**), Android fat mass loss (**c**), Gynoid fat mass loss (**e**) and Leg fat mass loss (**g**); It was also negatively correlated with total testosterone (**b**). Total testosterone changes were negatively associated with Android fat mass loss (**d**), Gynoid fat mass loss (**f**) and LNIR changes (**h**)

Gender analyses revealed that there was a higher percentage of sUA reduction in the HUA group compared to the NUA group for female patients (*P <* 0.05). In addition, the percentage of sUA reduction in the female HUA group was more significant than the percentage of sUA reduction in male HUA group (*P <* 0.001) (Fig. 1). In the male HUA group, there was a positive correlation between ΔsUA and ΔBW, ΔBMI, ΔNC, and ΔHC (*r* = 0.618, 0.653, 0.716, and 0.501, respectively; *P <* 0.05 in all cases). Moreover, the ΔsUA was positively correlated with ΔGynoid fat mass, ΔAndroid fat mass, and ΔLeg fat mass, (*r* = 0.675, 0.551, and 0.712respectively; *P <* 0.05 in all cases), as well as with ΔLeg lean mass (*r* = 0.631, *P =* 0.009) (Fig. 4a, c, e, and g).

**Fig. 5:**
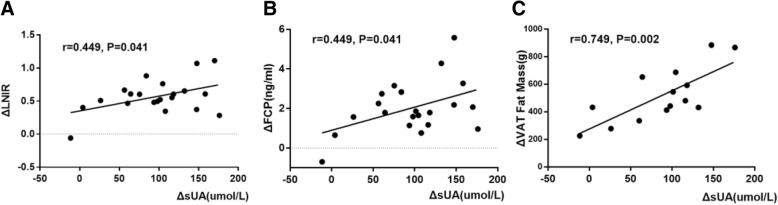
Correlations between ΔsUA and LNIR, FCP, and VAT fat mass changes in female HUA group. Correlations between changes in sUA and changes in LNIR, FCP, VAT fat mass in female HUA group. Changes in sUA were positively associated with changes in FCP (**b**), LNIR changes (**a**) and VAT fat mass loss (**c**)

In the female HUA group, ΔsUA was positively correlated with ΔFCP and ΔLINR (*r* = 0.449 and 0.449, respectively; *P <* 0.05 in both cases), and positively correlated with ΔVAT fat mass, ΔVAT fat volume, and ΔVAT fat area (*r* = 0.749, 0.749, and 0.747, respectively; *P <* 0.01 in all cases) (Fig. 5). These correlations were not observed in the female NUA group.

### The different effects of LSG on sex hormone levels in both genders, and the correlations with body fat distribution changes and insulin resistance

To further evaluate the effect of LSG on sex hormone levels in obese patients, we enrolled 71 premenopausal women (age < 50 years) and 48 men for analysis. The relevant sex hormone levels before and after surgery are shown in Table 3.

**Table 3:** Sex hormone levels at baseline and 6 months after LSG

	Male(*n* = 48)				Female(*n* = 71)			
	NUA group(*n* = 13)		HUA group(*n* = 35)		NUA group(*n* = 23)		HUA group(n = 48)	
	0 month	6 months	0 month	6 months	0 month	6 months	0 month	6 months
E2 (nmol/L)	0.13 ± 0.06	0.12 ± 0.09	0.13 ± 0.06	0.11 ± 0.04	0.27 ± 0.23	0.56 ± 0.33	0.17 ± 0.11a	0.27 ± 0.21a
TT (nmol/L)	10.14 ± 5.88	17.50 ± 9.54**	7.07 ± 3.27a	14.56 ± 3.84**	1.19 ± 0.68	0.89 ± 0.35	1.35 ± 0.88	0.92 ± 0.55*
P (nmol/L)	1.75 ± 0.64	2.11 ± 0.94	2.12 ± 1.17	2.06 ± 1.09	7.31 ± 16.45	6.16 ± 9.75	3.99 ± 7.32	8.95 ± 14.53
FSH (mIU/ml)	5.34 ± 3.47	5.72 ± 4.14	3.81 ± 11.51	4.05 ± 2.05	5.13 ± 2.88	4.76 ± 1.65	6.01 ± 7.58	4.79 ± 1.87
LH (mIU/ml)	6.86 ± 3.87	6.88 ± 3.37	4.47 ± 1.60	4.45 ± 1.42	6.14 ± 3.17	7.15 ± 4.21	6.71 ± 4.56	6.66 ± 5.27
E/T	12.35 ± 7.09	5.37 ± 2.23*	33.82 ± 47.20a	8.12 ± 3.53**	0.36 ± 0.54	0.69 ± 0.45	0.17 ± 0.15	0.41 ± 0.41*

Data presented as means ± SD; Compare HUA group to NUA group at baseline, ^a^ *P* < 0.05, ^b^ *P* < 0.01; Compare group after 6 months to baseline *P < 0.05, **P < 0.01

The baseline total testosterone (TT) levels in the male HUA group were lower than the baseline TT in the male NUA group (*P <* 0.05). At 6 months after surgery, the TT levels in the male HUA and NUA groups were both significantly higher than the TT levels in the preoperative male HUA and NUA groups (*P <* 0.01). The estradiol/total testosterone (E/T) ratios were significantly decreased in both male HUA and NUA groups (*P <* 0.05). Correlational analyses on postoperative sex hormone changes, uric acid changes, and fat distribution changes revealed a negative correlation between ΔsUA and ΔTT after LSG in the male HUA group (*r* = − 0.517, *P =* 0.040) (Fig. 4b). At the same time, ΔTT was negatively correlated with ΔTotal fat mass, ΔLimb fat mass, ΔTrunk fat mass, ΔGynoid fat mass, and ΔAndroid fat mass (*r* = − 0.816,-0.774, − 0.696, − 0.703, − 0.777, respectively; *P <* 0.01 in all cases) (Fig. 4d, f); however, there was no correlation with ΔVAT fat mass (*P >* 0.05). Interestingly, there was a negative correlation between ΔTT and ΔLNIR in the male HUA group (*r* = − 0.625; *P =* 0.010) (Fig. 4h). These correlations were not observed in the male NUA group.

The effects of LSG on sex hormone levels in women were different from the effects of LSG on sex hormone levels in men. The baseline estradiol (E2) levels in the female HUA group were lower than E2 levels in the female NUA group (*P <* 0.05). There was an increasing trend in E2 levels for female patients at 6 months after LSG, but the difference was not statistically significant (*P >* 0.05). The TT levels decreased in women after surgery, and postoperative TT levels were significantly lower than preoperative TT levels in the HUA group (*P <* 0.05). The E/T ratio in the HUA group increased after surgery (*P <* 0.05). There was a negative correlation between ΔE2 and ΔHOMA-IR in the female HUA group 6 months after LSG (*r* = − 0.585; *P =* 0.017).

## Discussion

Epidemiological data suggest that hyperuricemia is closely related to obesity [5–7]. In a 10-year follow-up study in a Canadian population that included black and white subjects, Rathmann et al. found that sUA levels increased gradually with increasing BMI [8]. Chen Mingyun et al. [9] published a study on 2962 patients with type 2 diabetes that also showed a gradual increase in the prevalence of obesity with increasing sUA quartiles. LSG can effectively reduce the body weight of obese patients while improving hyperuricemia [10]. Romero-Talamás et al. [11] studied 99 patients with gout and comorbid obesity who subsequently underwent metabolic surgery. They found that 13 months after surgery, the number of gout attacks decreased from 23.8 to 8.0%, and the average sUA levels of the patients also significantly decreased. In the 128 obese patients who underwent LSG in the present study, the average sUA level 6 months after surgery was lower than that before surgery. In addition, the decrease in sUA levels in patients who had hyperuricemia before surgery was more pronounced than in the NUA control group which is an observation in line with previous studies [12]. Intriguingly, gender differences were shown regarding the improvement of sUA after LSG. At 6 months after LSG, there was a significant reduction in sUA in the female HUA group, whereas no significant reduction in sUA was observed in the male obese patients 6 months after LSG.

Body fat distribution is often abnormal in patients with obesity. The elevated amount of body fat and abnormal body fat distribution not only causes disorders in lipid metabolism that may lead to insulin resistance, but is also correlated with oxidative stress and chronic inflammation in the body of obese patients. Bariatric surgery has been shown to improve glucose and lipid metabolism and lower body fat content and can also significantly improve body fat distribution. In our study, the body composition measurements in obese patients 6 months after LSG showed that LSG reduced the fat and lean masses in various body parts (including the head, neck, limbs, trunk, android region, and gynoid region) of the obese patients. The reduction in fat mass was more pronounced than the reduction in lean mass in all body parts, and the reduction in body fat was mainly due to reduced fat mass in the trunk. Android and gynoid are used to represent 2 main types of sex-specific fat distributions [13]. Compared to the male NUA group, the decrease in fat mass in the male HUA group 6 months after LSG primarily originated in the limbs and gynoid region, and the decrease in VAT fat mass in the HUA group was not as obvious as in the NUA group. The fat mass changes in various body parts of women appeared to decrease to greater extents in the HUA group than in the NUA group, but the difference was not significant.

We further evaluated the effects of LSG on uric acid and improvement of body fat distribution. We found that there was a close relationship between sUA changes and body fat distribution, and that their relationships displayed gender differences. Although the reduction in sUA levels in men was not as significant as in women 6 months after LSG, the improvement in sUA level in men was nonetheless positively correlated with BW, BMI, and HC, and positively correlated with the reduced fat distribution in the android region, gynoid region, and the legs. The reduction in sUA levels in male patients, which might rely on the decrease in body weight after surgery, was not correlated with changes in VAT. Meanwhile, our assessment of male sex hormone levels showed that LSG increased the TT levels in male obese patients, an observation that corroborated previous studies [14]. The improvement of postoperative TT levels in the male HUA group was negatively correlated with the decrease in sUA, and negatively correlated with the decrease in fat mass in the android and gynoid regions. These results suggested that the improvement of sUA levels in male obese patients after LSG might be related to the improvement in sex-specific fat distribution, in which testosterone might play a role in the regulatory process. The testosterone levels decrease as the amount of body fat increases in obese men. This may be due to the increased aromatase activity in the adipocytes in obese men that results in an increased conversion of androgens to estrogens, leading to an imbalanced estrogen/androgen ratio in the body [15, 16]. At the same time, previous studies have found that [17] testosterone is closely related to hyperuricemia. In male patients with gout, there is a concurrent reduction in the synthesis of testosterone and E2. It is speculated that the elevated sUA levels may affect hypothalamic hormone secretion and lead to decreased gonadotropin production, resulting in the reduction of testosterone and E2 synthesis. Our study suggests that the improvement of sUA after LSG in obese men is correlated with the improvement in sex-specific fat distribution and elevated TT levels. Though the specific mechanistic relationships remain unclear. The situations in obese female patients are different. The sUA levels in the female HUA group decreased 6 months after LSG, and were positively correlated with the reduction of VAT. This effect was independent of the decrease in body weight but was closely related to the amelioration of insulin resistance. Previous studies have also found that gender differences exist in the relationship between sUA and metabolic syndrome. A 5.4-year prospective study on 3857 Chinese patients published in Yang et al. [18] has shown that after adjusting for age, triglyceride levels, HDL-C, fasting blood glucose, and waist circumference, the baseline sUA levels and metabolic syndrome were more closely related in women than in men. In the present study, the improvement of sUA levels in female patients with hyperuricemia after LSG was more pronounced than in men. Furthermore, the decrease of uric acid was closely related to the reduction of VAT fat mass and amelioration of insulin resistance. The increased E2 levels, decreased TT levels, and increased E/T ratio in obese female patients 6 months after LSG might be related to sUA improvement. Epidemiological studies have shown that the difference in hyperuricemia risk in men and women is due to the difference in estrogen levels. Estrogen is an effective uricosuric agent [19]. Additionally, estradiol can affect fat metabolism and fat distribution in women. For example, fat accumulation in the abdominal cavity become more pronounced as estrogen levels in postmenopausal women decrease, which leads to abdominal obesity. LSG can reduce sUA levels and improve body fat distribution, and the latter 2 are closely related and display gender differences. Although the mechanisms underlying these observations are still unclear, the differences in sex hormone levels may partially explain the effects.

Interestingly, the drop in plasma insulin levels was larger than the drop in plasma glucose levels after surgery, and the postoperative decrease of insulin levels in the HUA group was not as significant as that in the NUA group. HOMA-IR and FGIR are sensitive indicators of insulin resistance in the body. Decreased HOMA-IR and increased FGIR after LSG indicates that surgery could improve insulin resistance in obese patients. However, the improvement of HOMA-IR and FGIR levels in the HUA group was not as significant as that in the NUA group, suggesting that hyperuricemia might be an adverse factor for improvement of insulin resistance post-LSG. There are several potential reasons for this. First, metabolic disorders are more severe in obese patients with hyperuricemia. Recent studies [20] on the correlation between metabolomics and obesity etiology have found that metabolites are closely associated with BMI. The majority of the 49 BMI-associated metabolites increase with increasing BMI, including glucose and mannose, which has been recently highlighted as an important factor in insulin resistance. Of all the metabolites, the one most closely associated with BMI is uric acid, which could explain 16% of the variance in BMI. This study revealed that metabolic disorders in obese patients with hyperuricemia were more severe and more difficult to correct. Second, there is clinical evidence [21] that the level of sUA is closely related to the inflammatory state of obesity; higher levels of uric acid are associated with a greater inflammatory state. This might be the reason why metabolic disorders in obese patients with hyperuricemia are more difficult to correct than those obese patients with normal uric acid levels. Finally, there might be a bidirectional causal relationship between hyperuricemia and insulin resistance [22]. Hyperuricemia plays a role in the development of insulin resistance in obese patients and may affect the functions of endothelial cells, such as reducing the production and bioavailability of nitric oxide, thereby exacerbating insulin resistance. In obesity, uric acid can be converted into a pro-oxidant and can participate directly in the proliferation and oxidative stress of adipocytes. sUA stimulates reactive oxygen species production and increases nicotinamide adenine dinucleotide phosphate (NADPH) oxidase activity in mature adipocytes [23]. In addition, clinical evidence suggests that sUA levels are associated with the levels of various cytokines secreted by adipocytes [24, 25]. Uric acid may act as a novel inflammatory factor involved in oxidative stress in adipocytes, inducing chronic inflammation and insulin resistance in obese patients [21]. On the other hand, increased sUA may be associated with reduction in uric acid excretion induced by insulin resistance and increased uric acid synthesis caused by increased visceral fat accumulation [26–28].

As uric acid exerts a bidirectional effect on regulating metabolism and promoting oxidative stress, excessively high or low levels of uric acid are undesirable. LSG reduces body weight, improves metabolism, and reduces the excessively high sUA levels back to normal in obese patients. In addition, it exerts minimal effects on the sUA levels in patients with normal baseline sUA, and its metabolic regulatory role is more pronounced in patients with hyperuricemia. We believe that the different effects of LSG on sex hormones in men and women lead to the differences in the improvement of fat distribution. The improvement of sex-specific fat distribution in men and VAT in women are associated with the differences in the reduction of sUA levels in men and women, respectively. Insulin resistance may serve as an important link between improved fat distribution and sUA levels and plays an important role in the development of obesity and hyperuricemia [29, 30]. A previous study has shown a stronger correlation between sUA and insulin resistance in women [31], so we hypothesized that insulin resistance might be related to gender differences in changes of sUA level and fat distribution after LSG. At 6 months after LSG, sUA levels in the female HUA group decreased, and more patients benefited from VAT reduction and insulin resistance improvement. In contrast, there was no significant improvement in sUA levels in the male HUA group, which might be related to the insignificant improvement in VAT.

The purpose of this study was to investigate the effect of LSG on sUA levels and body fat distribution. Few studies have investigated the gender differences when studying the relationships between postoperative sUA levels and body fat distribution after bariatric surgery. The limitations of this study included small sample size, which might lead to sample selection bias. In addition, the follow-up time of this study was relatively short, and some metabolic data had yet to show statistical differences. Future studies should focus on expanding the sample size and extending the follow-up period to provide more comprehensive and accurate clinical evidence-based medical data regarding the efficacy and mechanism of bariatric surgery.

## Conclusions

This study showed that high sUA levels of obese patients improved 6 months after LSG surgery. Reduction of sUA after LSG was correlated with improved body fat distribution, and the relationships also displayed gender differences. Effects of sUA reduction by LSG in male patients with hyperuricemia might be related to improved sex-specific fat distribution and regulation of total testosterone levels. However, the effects in female patients with hyperuricemia were mainly related to reduced visceral fat and ameliorated insulin resistance. Uric acid might play an important role in metabolic regulation.

## Abbreviations

BMI: Body mass index
BW: Body weight
E2: Estradiol
FCP: Fasting serum C peptide
FINS: Fasting serum insulin
FPG: Fasting plasma glucose
FSH: Follicle-stimulating hormone
H: Height
HbA1c: Hemoglobin A1c
HC: Hip circumference
HDL: High-density lipoprotein cholesterol
HOMA-IR: Homeostasis model assessment insulin resistance index
HUA: High serum uric acid
LDL: Low-density lipoprotein cholesterol
LH: Luteinizing hormone
LNIR: Natural logarithm of insulin resistance
LSG: Laparoscopic sleeve gastrectomy
NC: Neck circumference
NUA: Normal serum uric acid
sUA: Serum uric acid
TC: Total cholesterol
TG: Triglyceride
TT: Total testerone
VAT: Visceral adipose tissue
WC: Waist circumference
WHR: Waist-hip ratio
